# Integrating Assessment for Social Disconnectedness Into Aging Services: Charting a Path Forward

**DOI:** 10.1093/geroni/igab046.138

**Published:** 2021-12-17

**Authors:** Alice Prendergast, Kristi Fuller

**Affiliations:** Georgia State University, Atlanta, Georgia, United States

## Abstract

Social disconnectedness poses a serious threat to the health and well-being of older adults. Although research demonstrates that social disconnectedness was prevalent among older adults long before the COVID-19 pandemic, the crisis has brought significant attention to this issue, as well as resources to address it. The crisis also shed light on the current gap in evidence and guidance on how best to assess for and address social disconnectedness, especially in practice settings. Researchers from the Georgia Health Policy Center conducted a review of existing assessment tools and processes for social disconnectedness and formulated recommendations for the Georgia Division of Aging Services (DAS) in November 2020. These recommendations involve the use of evidence-based assessments paired with person-centered counseling to address social disconnectedness among at-risk individuals. In this session, researchers from Georgia State University will define social disconnectedness, explain how it differs from related constructs, and discuss the necessity of a holistic approach to assessment; summarize the review and recommendations made to DAS; and present preliminary data from DAS’s initial implementation of the assessment process.

